# Supplementary material for: Exploring the Association of HLA Genetic Risk Burden on Thalamic and Hippocampal Atrophy in Multiple Sclerosis Patients
Source: Genes (Basel). 2022 Nov 17;13(11):2136. doi: 10.3390/genes13112136 (PMC9690825; doi:10.3390/genes13112136)
Supplement: Supplementary file 1 [file genes-13-02136-s001.zip › genes-2010292-supplementary/Supplementary files/Supplementary files caption.pdf]

Supplementary Table S1. Association statistic for the 29 investigated HLA MS susceptibility loci and the MRI metrics.

In the Supplementary Table it is reported the beta and the p-values of association for each HLA MS susceptibility locus with the five brain metrics according to the reference allele indicated in the A1 column. In bold are reported the SNP statistically associated at nominal level.

Abbreviations: Regarding A1 column: Pr=presence, Ab=absence, A=adenine, C=cytosine, G=guanine, T=thymine; WMV=white matter volume; GMV=gray matter volume; HippV=hippocampal volume; ThalV=thalamic volume; T2LV=T2 lesion volume.

Supplementary Table S2. Association statistic for the 10 investigated HLA alleles associated at $p<5x10^{-8}$ in Bian et al and the MRI metrics.

In the Supplementary Table it is reported the beta and the p-values of association for the 10 investigated HLA alleles associated at $p<5x10^{-8}$ in Bian et al with the five brain metrics according to the reference allele indicated in the A1 column. In bold are reported the SNP statistically associated at nominal level.

Abbreviations: Regarding A1 column: P=presence; WMV=white matter volume; GMV=gray matter volume; HippV=hippocampal volume; ThalV=thalamic volume; T2LV=T2 lesion volume.
